# Unraveling plant–microbe interactions: can integrated omics approaches offer concrete answers?

**DOI:** 10.1093/jxb/erad448

**Published:** 2023-11-09

**Authors:** Roy Njoroge Kimotho, Solomon Maina

**Affiliations:** Hebei Key Laboratory of Soil Ecology, Key Laboratory of Agricultural Water Resources, Centre for Agricultural Resources Research, Institute of Genetics and Developmental Biology, Chinese Academy of Sciences, Shijiazhuang 050021, China; University of Chinese Academy of Sciences, Beijing 100049, China; Elizabeth Macarthur Agricultural Institute, NSW Department of Primary Industries, Menangle, New South Wales 2568, Australia; University of Warwick, UK

**Keywords:** Integration, metabolomics, microbiome, omics, plant–microbe, rhizosphere

## Abstract

Advances in high throughput omics techniques provide avenues to decipher plant microbiomes. However, there is limited information on how integrated informatics can help provide deeper insights into plant–microbe interactions in a concerted way. Integrating multi-omics datasets can transform our understanding of the plant microbiome from unspecified genetic influences on interacting species to specific gene-by-gene interactions. Here, we highlight recent progress and emerging strategies in crop microbiome omics research and review key aspects of how the integration of host and microbial omics-based datasets can be used to provide a comprehensive outline of complex crop–microbe interactions. We describe how these technological advances have helped unravel crucial plant and microbial genes and pathways that control beneficial, pathogenic, and commensal plant–microbe interactions. We identify crucial knowledge gaps and synthesize current limitations in our understanding of crop microbiome omics approaches. We highlight recent studies in which multi-omics-based approaches have led to improved models of crop microbial community structure and function. Finally, we recommend holistic approaches in integrating host and microbial omics datasets to achieve precision and efficiency in data analysis, which is crucial for biotic and abiotic stress control and in understanding the contribution of the microbiota in shaping plant fitness.

## Teaser

A review of integrated omics approaches in the elucidation of plant–microbe interactions driving resilience to environmental stresses and shaping plant health, along with holistic solutions to their shortcomings.

## Introduction

Global food demand is anticipated to increase by 70% by the year 2050 due to the rapid increase in human population (FAO, 2019). This means that over the next 30 years, food production need to be significantly accelerated. For many decades, improved farming methods such as planting improved seeds derived from breeding techniques have delivered increased food productivity (Yu and Li, 2022; Zhang *et al.*, 2023). However, over recent years the rate of increase in food production has been declining (FAO, 2019; Zhu *et al.*, 2022). For instance, the current global annual growth rates of 1.1–1.8% for rice, maize, soybean, and wheat are expected to decline in coming years (Iizumi *et al.*, 2018). Such an impact has been driven by declining crop yields contributed by such factors as abiotic and biotic stresses, and geopolitical conflicts in various parts of the world (FAO, 2017; Arif *et al.*, 2020; Nasir *et al.*, 2022). For example, Ug99, a new wheat stem rust strain, is spreading aggressively across the globe helped by its strong immunity to most of the wheat resistance genes, thereby threatening global food security (Li *et al.*, 2019). Similarly, the global production of the Cavendish banana is threatened by highly virulent *Fusarium oxysporum* (TR4), which is spreading throughout the major banana-growing regions around the globe (Fones *et al.*, 2020). Further, maize lethal necrosis disease has had a tremendous negative impact on maize production in large parts of sub-Saharan Africa (Wamaitha *et al.*, 2018).

Global climatic changes are projected to continue to have adverse effects on current and future crop yields (Zhang *et al.*, 2022; Zhu *et al.*, 2022). Warmer temperatures in particular account for about 20% of the observed rise in plant disease occurrences globally (Raza and Bebber, 2022). Notably, a direct relationship has been identified between climate change and plant biotic stresses, for instance the locust outbreaks in sub-Saharan Africa in 2019 and 2020 (Salih *et al.*, 2020). Fones *et al*. (2020) highlighted this relationship by introducing a novel concept, the ‘disease triangle’, in which worsening climatic conditions are exacerbating plant pathogen migration and evolution, creating challenges for vector control and associated disease management. In addition, frequent heat waves are aggravating drought conditions and increasing desertification in different regions of the world (Eckardt *et al.*, 2023). According to the Aqueduct Water Risk Atlas (World Resources Institute, 2023), Europe, Asia, and Africa carry a medium to high risk of drought exposure. All these examples highlight the increased challenges facing global food security in the 21st century. The recent 2023 report on the state of food security and nutrition in the world by the Food and Agriculture Organization of the United Nations (FAO, 2023), paints a grim picture regarding the future of global food security. The report states that even if the 2020 Covid-19 pandemic or the war in Ukraine had not happened, the world would still be set to miss the second Sustainable Development Goal of zero hunger by 19.86% by the year 2030 (Yuan *et al.*, 2023). Further, the human population facing acute food insecurity rose from 137 million in 2019 to more than double (346 million) in mid-2022. Efforts to address the high global food demand have been associated with farming practices that result in the deterioration and degradation of the environment. This has further derailed the advances made towards Sustainable Development Goal 15 (Hossain *et al.*, 2020; Sasmito *et al.*, 2023). For example, A positive correlation has been identified between land degradation and poverty, especially in low-income countries in parts of Asia and sub-Saharan Africa (Barbier and Hochard, 2018). Further, studies have also shown that hunger, land degradation, starvation, and poverty are not independent events but are entwined (Barbier and Hochard, 2018). Taken together, these analyses describe the multiple unprecedented challenges that global food systems, environment, and climatic conditions are facing that must be addressed in sustainable ways. All these challenges have led to significant limitations in crop production. Therefore, failure to address these challenges in both the short and long term will have enormous consequences for food security, climate conditions, and environmental sustainability that will likely be felt for decades to come.

To address these challenges, an opportunity exists with regard to plant-associated organisms such as microbes and arthropods that have a significant influence on crop productivity (Bagchi *et al.*, 2014; Gruden *et al.*, 2020; Saad *et al.*, 2020). They are regarded as the plants’ ‘second genome’, inhabiting the rhizosphere, endosphere, rhizoplane, spermosphere, and phyllosphere (Trivedi *et al.*, 2020). Crop-associated microbial communities reinforce plant performance and fitness, and hence the need for additional studies to unravel the biological mechanisms involved in these interactions so as to harness their full potential (Carrión *et al.*, 2019; Trivedi *et al.*, 2020; Shalev *et al.*, 2022). Many studies have focused on harnessing the rhizosphere microbiota in sustainable crop production, and there has been profound success (Bender *et al.*, 2016; de Vries and Wallenstein, 2017). This is particularly timely given the world faces a plethora of challenges that hinder increased crop productivity: agricultural microbiomes potentially offer contributions to sustainable crop production (Box 1). A review by de Vries *et al.* (2020) highlighted the potential applications of rhizosphere microbiomes in the production of drought-resilient crops. The authors offered recommendations to quantify host and microbial traits focusing on food crops. They emphasized the importance of understanding plant–microbe interactions in response to droughts and recurring droughts to maximize crop yields. This is of paramount importance given that the number of countries affected by drought keeps on increasing and persistent droughts are becoming more severe (Funk, 2020).

Box 1. **Trends and challenges of current agricultural practices and proposed solutions involving plant–microbe associations**

Although agriculture has become more efficient at the global scale in recent decades, land degradation is emerging as a major impediment to food security. Globally, 34% of agricultural land is moderately to severely degraded due to anthropogenic factors such as deforestation, excessive fertilizer use, erosion, and mining (Hossain *et al.*, 2020). Moreover, a large percentage of the additional land available is unsuitable for agriculture (Da Silva, 2012). This means that we need to restore and utilize the available agricultural land more sustainably. Soil microbiota in association with plants may play a vital role in restoring degraded soils. Plant growth-promoting rhizobacteria, arbuscular mycorrhizal and ectomycorrhizal fungi, cyanobacteria, and nitrogen-fixing bacteria have been shown to directly or indirectly contribute to land restoration (Coban *et al.*, 2022).

The world is facing phosphorus challenges driven by an increase in food demand, phosphorus scarcity, and overuse of synthetic phosphorus fertilizers that leads to eutrophication (Cong *et al.*, 2020). Globally, rock phosphate (the main source of phosphorus) reserves are anticipated to be depleted in 50–100 years (Alewell *et al.*, 2020; Scholz and Wellmer, 2021). In addition to the decreasing rock phosphate reserves, increasing costs and uneven global distribution of the reserves have aggravated concerns (Filippelli, 2018; Alewell *et al.*, 2020). Loss of phosphorus from agricultural systems will significantly limit our food and feed production in the coming decades. Plant associations with microbes, for instance phosphate solubilizing fungi and bacteria and arbuscular mycorrhizal and ectomycorrhizal fungi, can significantly enhance the efficiency of phosphate solubilization and acquisition by crops (Wang *et al.*, 2013; Hiruma *et al.*, 2016; Cong *et al.*, 2020; Lidbury *et al*., 2021). Therefore, future studies should focus on crop-associated microbiotas and their roles in phosphate acquisition in crops whether at the genus, phylum, or inter-kingdom levels (Castrillo *et al.*, 2017).

The domestication process has led to breeding programs that have allowed the selection of more suitable and productive genotypes able to cope with climate change (Rasheed *et al.*, 2017; Yu and Li, 2022). However, despite these successes several challenges have been encountered, for instance reduced genetic diversity (Cieslarová *et al.*, 2011), challenges in understanding polygenic traits (Mondal *et al.*, 2021), and accumulation of unfavorable alleles (Gaut *et al.*, 2018), among others. Therefore, we propose implementing a holistic view of plant breeding to include the exploitation of plant–microbe multi-omic approaches that will facilitate a deeper elucidation of the plant–microbiome interactions (Porter and Sachs, 2020).

Globally, plant protection against phytopathogens heavily relies on two approaches: the use of fungicides and crop resistance breeding. However, the repeated use of fungicides has led to the development of resistance in most plant pathogens (Hahn, 2014). Therefore, novel plant protection approaches are needed to mitigate the effects of fungicide resistance, especially in a warming climate (Hahn, 2014; Corkley *et al.*, 2022). Harnessing the potential of plant-associated microbiota in plant defense has two main benefits: (i) recruitment of soil microbes as the first line of defense by plants against soil pathogens (Mendes *et al.*, 2011; H. Liu *et al.*, 2021) and (ii) systemic induction of plant immune responses against pathogens without detrimental effects to the plants (Pieterse *et al.*, 2014; Lee *et al.*, 2021).

Crops interact with many microbes and this supports them in dealing with abiotic and biotic stresses, a vital trait that should be deeply studied. Moreover, beneficial soil microbiota should be thoroughly exploited as modern agriculture moves into the second green revolution (Nerva *et al.*, 2022b; Trivedi *et al.*, 2021). Applying a holistic view in our current management practices that includes plant microbe multi-omic approaches will help us identify valuable traits that can be integrated into current breeding programs (Trivedi *et al.*, 2020; Nerva *et al.*, 2022b).

The microbial diversity of different plant organs has been widely studied using high-throughput molecular approaches (Abdelfattah *et al.*, 2016; Yuan *et al.*, 2018; Regalado *et al*., 2020; Matsumoto *et al.*, 2021). In particular, root exudation and morphology have a major effect on the chemical and physical properties of the rhizosphere (Lundberg and Teixeira, 2018; Yuan *et al.*, 2018; Chai and Schachtman, 2022). Different plant species associate with distinct microbial communities and this shapes their rhizosphere microbiomes, especially when they are grown in the same soil (Ofek-Lalzar *et al.*, 2014; Fitzpatrick *et al.*, 2018). Moreover, different crop genotypes are hosts to different microbial communities (Zhang *et al.*, 2020; Cordovez *et al.*, 2021). For instance, vigorous-growing genotypes harbor a more functionally diverse microbiome that contributes to better plant performance (Wagner *et al.*, 2016; Brown *et al.*, 2020; Leopold and Busby, 2020). This suggests a high likelihood of co-evolution within crops and their beneficial microbes during their evolutionary interactions (Mousa *et al.*, 2016; Abdelfattah *et al.*, 2022). However, many of these studies applied a single omics technology resulting in a limited understanding of the complexities and opportunities within the plant microbiome. There is therefore an urgent need to go beyond one-dimensional taxonomic and functional studies into integrative studies. Realistically, multi-omics adoption is not likely solely to be sufficient to increase food production to satisfy the global population, but successful integrative omics investigations will contribute to deeper elucidation of biological functions within the plant microbiome. For instance, the use of integrated omics approaches can contribute to microbiome engineering of synthetic communities with growth promotion and protective traits specific to individual crops, leading to increased food supply and a decrease in the use in agricultural systems of harmful chemicals in fertilizers, herbicides, fungicides, and insecticides.

There are recent successful studies that have demonstrated how the integration of multi-omics techniques has been instrumental in unraveling plant-associated microbiomes and their potential to contribute to improved crop productivity. For example, Yu *et al.* (2021) used a combination of transcriptomics, metagenomics, and metabolomics to show that genotype-dependent root transcription changes in maize, defined strong differences in root rhizosphere microbiomes between two inbred lines. This study found that transplanting inbred line LH93 into soil previously used to grow inbred line 787 significantly increased the shoot biomass of line LH93; however, when inbred line 787 was transplanted into the soil previously used to grow inbred line LH93, substantial growth inhibition was observed. These findings revealed that plants cultivated in favorable conditions can recruit beneficial microbes during biotic or abiotic stress or nutrient deprivation conditions, a phenomenon referred to as the ‘cry for help’ strategy (Hu *et al.*, 2018; H. Liu *et al.*, 2021). The microbial communities’ contribution may include increased productivity (de Vries and Wallenstein, 2017; de Vries *et al.*, 2020) and overall plant health (Xie *et al.*, 2019; Pratama *et al.*, 2020). Further, Howe *et al.* (2023) used an integrated approach to uncover the roles of phyllosphere microbiomes in perennial grass species. They used integrated metatranscriptomics, metagenomics, metabolomics, and metataxonomics to decipher the main microbial communities that are responsive to host cues regarding environmental stresses. Poorly annotated biosynthetic pathways, for instance those for non-ribosomal peptides and terpenes, were also detected. This study is timely considering most multi-omics studies have focused almost entirely on rhizosphere microbiome interactions (Turner *et al.*, 2013; Zancarini *et al.*, 2021; Chen *et al.*, 2023), leaving out the phyllosphere, which is the largest microbial habitation in the world (Peñuelas and Terradas, 2014). This work showed the power of integrating omics, as compared with using a single approach, by capitalizing on the distinct information produced by different omics approaches. Elsewhere, a comprehensive study by Ichihashi *et al.* (2020) showed the potential of integrated omics within the agroecosystem. Using integrated omics analysis, complex interactions between microbial communities and soil metabolic and mineral components were revealed. Within the multi-omics data network, they found one node corresponded to plant productivity, and thermophilic bacteria (including *Thermaceae* and *Paenibacillaceae*) and soil organic nitrogen were among the crucial components. These findings revealed the predictive power of integrated omics approaches in detecting multilevel associations between plants, microbes, and soil and in identifying crucial components involved in the functioning of agricultural ecosystems. These examples illustrate the potential of integrated multi-omics approaches to increase our understanding of complex plant microbiome functioning in addition to supporting the development of novel solutions to today’s problems. Therefore, it is critical to develop and adopt integrative omics investigative approaches for studying plant–microbe interactions. This review provides an in-depth overview of the currently available omics approaches used to study plant microbiome interactions. It also demonstrates the importance and shortcomings of using integrated omics approaches toward improved crop productivity, and proposes holistic solutions to those shortcomings.

## Overview of current gaps in crop microbiome research

Knowledge of plant-associated microbiomes and their impact on food crop productivity and general plant health is limited (Howard *et al.*, 2017; Gamalero *et al.*, 2022). This necessitates a holistic understanding of the mechanistic and functional aspects of interactions between plants, microbes, soil, and agricultural management practices, to fully realize the benefits of crop microbiomes in agricultural productivity. Plant-associated microbial species may coexist with or colonize host plants, developing different lifestyles from mutualist to pathogen depending on the environment (Hacquard *et al.*, 2016; Hiruma *et al.*, 2016; Tian *et al.*, 2020). Therefore, characterization of plant microbiome members beyond identification, to functionally characterize individual species, will necessitate the development of new high-throughput techniques. Adopting additional microbiome engineering has the potential to improve nutrient efficiency, abiotic and biotic stress tolerance, and increase productivity in crops (Arif *et al.*, 2020; Albright *et al.*, 2022). This will necessitate significant improvements in the characterization techniques currently used and require experimental approaches that are dominated by census-based illustrative approaches (Lebeis, 2015; Busby *et al.*, 2017). Such improvements will enable the characterization of the molecular and chemical signals involved in the recruitment of beneficial microbes live and *in situ*. Also, the understanding of structural and functional responses of core microbiota to agronomic practices in different crops will contribute to addressing some of the knowledge gaps.

**Fig. 1.**
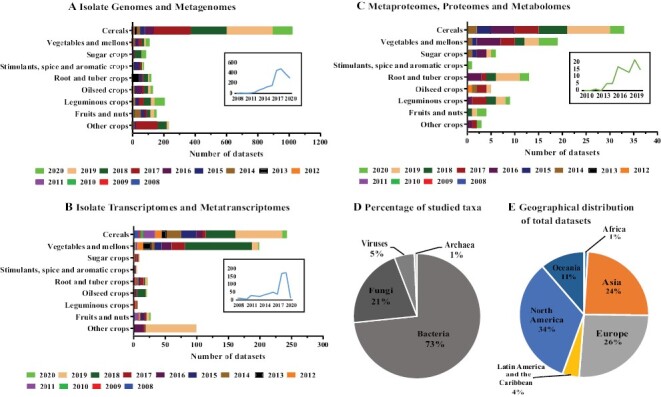
Total number of omics datasets generated from plant–microbiome studies each year. (A–C) The number of genomes and metagenomes (A), transcriptomes and metatranscriptomes (B), and metaproteomes, proteomes, and metabolomes (C) from different crop groups between 2010 and 2020. Data were obtained from NCBI BioProject, Integrated Microbial Genomes, MassIVE systems, and ProteomeExchange. The insets show the cumulative number of different datasets for all crops throughout the years. (D) Percentage of studied taxa in crop microbiome studies between 2010 and 2020. (E) Geographical distribution of total studied taxa between 2010 and 2020.

Over the past 30 years, there has been a 20-fold increase in published scientific articles describing the plant microbial communities’ complexities and symbiotic associations (Brunel *et al.*, 2020). This has been driven by technological advances such as high throughput sequencing (HTS: metatranscriptomics, metagenomics, bioinformatics) and gene editing, which have reinforced research into plant health and plant–microbe interactions (Box 2) (Levy *et al.*, 2018; Depuydt *et al.*, 2023). Despite this growth, research involving food crop microbiomes (rice, maize, wheat, potatoes, beans, cassava, etc.) remains incomplete (Busby *et al.*, 2017). To understand what limits our knowledge of crop microbiomes and decipher the integrative analysis potential of the datasets, we summarized the total number of research themes for the major food crops microbiomes. A literature search was done for each of the major crop categories based on the FAO’s Indicative Crop Classification, Version 1.1 (Da Silva, 2012; FAO, 2020). This search was done using the queries ‘plant name’, ‘microbial communities’, and ‘plant name’ microbiome’ on the NCBI BioProjects, Integrated Microbial Genomes (JGI), MassIVe systems, and Proteome Exchange databases. The literature search was carried out between March 2019 and December 2020, and we highlighted projects and metadata from research carried out since 2008 for the analysis. From these studies, we extracted the main topics and information on each crop using a classification based on genomes and metagenomes (Fig. 1A), transcriptomes and metatranscriptomes (Fig. 1B), and metaproteomics, proteomes, and metabolomes (Fig. 1C). Overall, a limitation in scale was noted in the literature analysed; for instance, approximately 70% of the literature reviewed had a single sampling time point, and 87% of the studies concentrated on one crop species (representing 55% of total crops studied) (Fig. 1).

Box 2. **A subset of omics techniques used to characterize plant hosts and their microbiota and challenges encountered in these approaches**

**Exometabolomics** is an emerging field in metabolomics, recently gaining focus in plant microbiome interaction studies (Zancarini *et al.*, 2021). Traditional metabolomics methods and analyses have emphasized what happens and what is produced inside the host and or microbial cells. Exometabolomics is consequently gaining prevalence as a solid approach to obtaining and processing rich phenotypic datasets in plant microbiome studies (Jacoby *et al.*, 2018; Jacoby and Kopriva, 2019).

**Metagenomics** can be used to analyse the functional potential and to obtain the taxonomic composition of microbial communities (Ye *et al.*, 2019; Kress *et al.*, 2022). However, these methods are expensive and require large amounts of microbial starting material. Additionally, most techniques do not discriminate between transient DNA and live microbes (Cheng *et al.*, 2019).

**Metataxonomics** targets highly conserved genes, for instance fungal 18S rRNA and bacterial 16S rRNA (Edgar, 2018). These approaches are cost-effective and have helped researchers characterize microbial taxa at the genus level. However, most protocols used in metagenomics are very sensitive to contaminants and cannot differentiate DNA from live or dead microbes (Quince *et al.*, 2017).

**Pangenomics** is a new approach that aims at understanding the genomic diversity of individual species in both plant hosts and microbial communities (Golicz *et al.*, 2020). This approach is timely considering the evolution of reference genomes from single organisms to the pangenome (Bayer *et al.*, 2020; Golicz *et al.*, 2020). The number of reported plant and microbial pangenomes keeps rising, in addition to the improved ability to build high-resolution pangenomes capturing all variations (Golicz *et al.*, 2020; Lei *et al.*, 2021). Therefore, the integration of microbial and host pangenomes promises to give more resolution to future plant microbe studies. However, some limitations still exist; for instance the breeding history of crop plants can affect their accessory gene content (Golicz *et al.*, 2020). Also, these high-resolution pangenomes will demand the development of novel tools (Lei *et al.*, 2021).

In **culturomics** the growth of microbial communities is tested in different media conditions by using high throughput culture approaches, followed by sequencing and mass spectrometry to identify growing microbial communities (Demirjian *et al.*, 2023; Mapelli *et al.*, 2023). Culturomic approaches are able to detect living microbes in addition to detecting minority populations. Coupled with other omic approaches, culturomics can help identify novel mechanisms in plant–microbe interactions (Demirjian *et al.*, 2023). However, this approach is prone to contamination and not ideal for non-culturable organisms (Sarhan *et al.*, 2019).

**Metaproteomics and metatranscriptomics** help in the identification of active functions in host plants and microbial communities (Turner *et al.*, 2013; Alkan *et al.*, 2015; Marquez *et al.*, 2019). These approaches can be used to track and analyse the global expression of genes and their functions. However, limitations include high costs in addition to the quick responses of microbiota to environmental fluctuations during experimental set-ups and in the process of handling collected samples.

Omics approaches are able to give a comprehensive representation of different biological systems, but the high number of variables increases the chance of detecting false positives. Compared with these individual analyses, the integration of different omics approaches increases the complexities of analysing plant microbiome datasets. The development of analytical methods to integrate multiple datasets still remains a challenge. Techniques such as canonical correlation analysis offer powerful platforms that can be used to integrate multi-omic data and have been successfully applied in different fields (Witten *et al.*, 2009; Helmer *et al.*, 2020, Preprint). In addition, recent advances in artificial intelligence offer a robust alternative that increases accuracy by reducing human error.

The results revealed that researchers were mainly interested in genomic and metagenomic studies (Fig. 1) with the least research being conducted on crop microbiome metabolomics (Fig. 1). Additionally, overall crop microbiome research was mostly focused on cereal and vegetable crops (Fig. 1). Further, studies on crop microbiomes were generally restricted to a phylum-level approach with the bacterial phyla receiving special attention (73% of all research analysed bacterial communities), while intricate interactions between archaea, bacteria, fungi, viruses, and the crop host remained inadequately studied (Fig. 1D). It may be that restriction to a phylum-level approach is a technological limitation, which may persist in the foreseeable future unless the quality of databases is improved. In terms of geographical distribution, our analysis found that available research exhibits strong regional localization of the studied datasets, whereby approximately 34% of total studies involved North America, with only 4% and 1% of total datasets available from Latin America–Caribbean and Africa, respectively (Fig. 1E). A recent review by Brunel *et al.* (2020) found a significant increase in rhizosphere metagenome datasets published each year from all the continents. It is notable that the increasing trend was briefly disrupted by the Covid-19 pandemic (Fig. 1). The above analyses revealed the current achievements, challenges, and gaps in understanding food crop microbiomes. Given the diverse nature of microbiomes described in similar food crops grown in different geographical regions, more investment is needed to close the research gap between the developed and the developing world. This will enhance understanding of microbiomes and elucidate the key mechanisms of how crops recruit and modulate their microbiomes in different environmental conditions to improve food productivity.

## Potential impacts of plant microbiomes

Beneficial plant microbes have enormous potential in boosting plant health and fitness, thereby improving yields and productivity. A report by the FAO (2019) emphasized the importance of microbiome research and its role in maintaining sustainable food systems and healthy diets. The report also highlighted the role of the microbiome within production systems to minimize degradation of the natural environment. Studies have highlighted the potential of plant-associated microbiota to improve plant defense, stress tolerance, and nutrient acquisition without or with less environmental impact (Finkel *et al.*, 2017). For instance, the presence or absence of several genes in either the microbes (e.g. genes that promote plant stress responses) or the plant host (e.g. pathogenesis-related proteins which protect host plants from pathogens) can have a significant effect on crop–microbe interactions (Zhang *et al.*, 2020; Hou *et al.*, 2021). Moreover, although plant microbiomes from different plant species are phylogenetically diverse, some encode matching proteomes and gene functions (Lambais *et al.*, 2017; Levy *et al.*, 2018). These matching proteomes and gene functions are vital to the survival traits required by microbes to thrive on different surfaces (roots, leaves, seeds) (Lambais *et al.*, 2017). As such, many genes involved in plant colonization are conserved in different bacterial taxa and plant-associated oomycetes and fungi (Levy *et al.*, 2018).

With the current changes in climate, it is vital to improve the resilience and production of crops under diseased, nutrient-poor, and abiotic stress conditions. Understanding the underlying mechanisms of plants growing in hardy areas, for instance deserts and nutrient-poor soils, can help ensure food security. Using multi-omics approaches, researchers can identify not only plant growth-promoting microbes near plant roots but also the positively selected genes associated with the essential processes of plant fitness and survival (Huot *et al.*, 2014; Hiruma *et al.*, 2016). For instance, Eshel *et al.* (2021) highlighted the potential of transcriptomics and by extension integrated plant microbiome omics. The study obtained 32 plant species’ transcriptomes from the Atacama Desert and analysed their microbiomes using metabarcoding sequencing. This study uncovered that the top genes expressed in these plant species were enriched in metabolism, energy production, and stress responses. Additionally, they also found that the main root-associated microbes were associated with nitrogen fixation and growth promotion. This research presented an opportunity to understand plant–microbe interactions. Genes associated with plant survival in extreme desert conditions were discovered in addition to nitrogen-fixing and growth-promoting microbes.

Despite the advances of such studies, it is still not clear how complex plant–microbiome interactions occur mainly because the critical beneficial microbes and host association are usually studied independently. This limitation may have been contributed by plant–microbial interaction studies being built on culture-dependent approaches. Nevertheless, the remarkable information exposed by HTS approaches over the past decades has revolutionized understanding of plant–microbe interactions (Fitzpatrick *et al.*, 2020). Therefore, employing such advances in HTS and gene editing approaches can unravel the complex plant–microbe interactions. These approaches have been used to study the detrimental effects of crop microbiomes and the origin and evolution of plant pathogens. For instance, Karasov *et al.* (2018) utilized a combination of metagenomics, 16S amplicon sequencing, pan-genome analyses, and phylogenetics to study the evolution of *Pseudomonas* pathogens in Arabidopsis. This study revealed a particular *Pseudomonas* lineage that diverged about 300 000 years ago (Karasov *et al.*, 2018). In addition, several genetically similar pathogenic sub-lineages showed different disease phenotypes and gene content. More importantly, the authors found that in wild plant populations, a single abundant lineage is not able to assume control of the host population, as opposed to in domesticated plants. Moreover, the application of multi-omics has also revealed complex biological mechanisms between different plant traits, microbiomes, minerals, and soil metabolites. For example, Zhalnina *et al.* (2018), used a combination of exometabolomics and genomics to understand the plant root exudation patterns controlling specific microbial communities from two distinct *Avena* species. Similarly, the integration of metabolomics, metatranscriptomics, and 16S rRNA sequencing depicted an increased activity of bacterial ATP-binding cassette (ABC) transporter genes, which altered metabolism, consistent with shifts in community composition in the sorghum root microbiome under drought stress (Xu *et al.*, 2018).

Understanding plant microbiome interactions can be important because (i) many vital traits in crops are influenced by host–microbe interactions, and (ii) plants can control microbiome assembly and the corresponding abundance of the microbial components via the release of root exudates (Hu *et al.*, 2018; Lundberg and Teixeira, 2018; Sasse *et al*., 2018). Further, volatile organic compounds released by plant roots have been identified as key drivers of plant microbiome assembly (Khashi u Rahman *et al.*, 2019). In this work, we will review the different approaches that have been employed to integrate microbiome data and plant omics datasets. Integrated omics techniques, their corresponding bioinformatics software, and statistical approaches have been discussed elsewhere. This work also analyses the current and future omics approaches and techniques that can be deployed in studying plant-associated microbiomes to increase plant fitness. Additionally, we highlight the knowledge gaps and technical limitations that hinder the full development of the field and suggest holistic approaches to addressing these concerns.

## Integrated omics at the interface of plant microbiomes and abiotic/biotic stresses

**Fig. 2.**
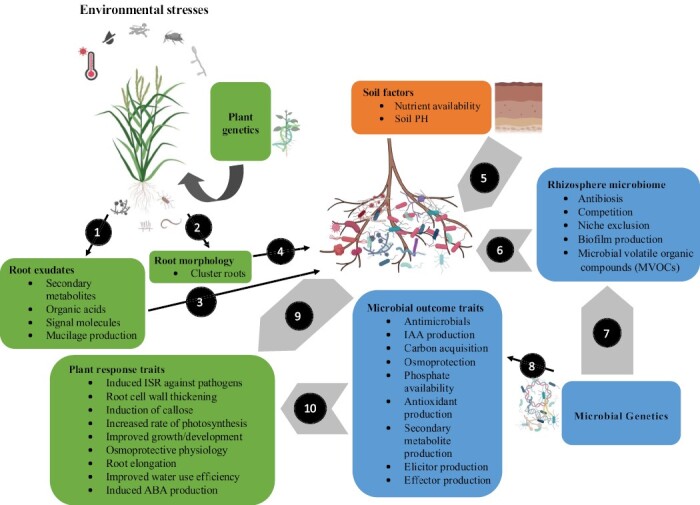
Application of omics in the recruitment and assembly of plant-associated microbiomes during biotic and abiotic stress conditions. Microbial composition in different plant organs is influenced by different abiotic and biotic factors (for instance high temperature, drought, pests, and pathogens), together with soil factors (soil pH and soil nutrients). Plant genetics (genomics) is a key factor influencing plant exudation patterns (1) and root morphology (2). Subsequently, rhizosphere microbiome assembly is strongly regulated by root exudates (3), and root structure (4). Soil factors are a major driver of soil microbiome composition and influence the recruitment of beneficial microbes by plants (5). Furthermore, microbe–microbe interactions that shape the rhizosphere microbiome can be studied using metabolomics and metatranscriptomics to uncover antimicrobial and/or probiotic molecules and genes involved in competition by different microbes (6). Microbial genetics is a key factor that influences how the soil microbiome is attracted to and utilizes different metabolites released by plant roots (7). Using host genomics, metabolomics, and transcriptomics together with microbial metagenomics we can understand key factors influencing plant exudation patterns that drive plant recruitment of specific microbial communities under environmental stresses (8). Using metagenomics and metatranscriptomics, researchers have been able to uncover genes involved in biofilm production, effector production, chemotaxis, and carbon acquisition that contribute to plant root colonization and the subsequent endophytic lifestyle inside the plant. Once established, the root associated microbiome offers benefits to the plant in the form of direct mechanisms (9) or indirect mechanisms (10), which helps the plants to be more tolerant of environmental stresses. ABA, abscisic acid; IAA, indole-3-acetic acid; ISR, induced systemic resistance; VOCs, volatile compounds.

Despite HTS omics advances, our understanding of the molecular mechanisms behind crop microbiomes in response to abiotic and biotic stresses is still limited (Trivedi *et al.*, 2022). One remedy to bridge this gap would be increasing the number of studies utilizing integrated omics approaches. We suggest using multi-omics approaches in combination with plant immune output, stress hormone profiling, and machine learning and network analysis to link the plant stress response and multi-omics datasets. This will help in developing models of the biological processes at the center of plant–microbe associations in different environmental stress conditions. This is because plant immunity and hormones largely influence the assembly and recruitment of plant-associated microbiomes in addition to influencing plant–microbiome–environment associations (Carrión *et al.*, 2019; Chen *et al*. 2020; Tian *et al.*, 2020; Lee *et al.*, 2021). Moreover, future studies should focus on investigating microbial roles in plants to understand abiotic and biotic stress responses (Fig. 2), which have been found to protect against desiccation and death (Martiny *et al.*, 2013; Xu *et al.*, 2018). Microbial traits employed for stress tolerance depend on the type of stress exposure; for instance, in high salinity conditions microbes utilize different approaches to maintain osmotic balance (Malik *et al.*, 2020). This has been observed within the co-evolution processes of *Actinomycetes* and fungi, crops, and crop root endophytes (Mousa *et al.*, 2016; Albright and Martiny, 2018). For instance, Gonin *et al.* (2023) utilized omics approaches and showed the Arabidopsis root microbiome influenced auxin-independent root branching. As such, mining for particular gene expression levels can assist in identifying unknown traits (Arif *et al.*, 2020; Ke *et al.*, 2021; Wilhelm *et al.*, 2023), and this can be adapted to predict crop microbiome dynamics. In particular, a study by Lozano *et al.* (2021) revealed saprophytic, mutualistic, and pathogenic fungal drought response was highly dependent on root trait adjustment to the conditions. Omics technologies can also obtain precise and meaningful information from plant microbiome studies focusing on environmental stress responses. This has been shown through a biological network inference where Carrión *et al.* (2019) found that after a fungal infection, *Flavobacteriaceae* and *Chitinophagaeceae* were significantly enriched in the endosphere of sugar beet. Additionally, chitinase genes, gene clusters encoding polyketide (PKSs), and non-ribosomal peptide synthetases (NRPSs) were enriched in the root endophytic microbiome (Carrión *et al.*, 2019).

Plant–microbe trait-based omics offer a better alternative to traditional species-based studies because they emphasize functional traits in plants and microbes rather than microbial species identities. For instance, Hou *et al.* (2021) found a link between microbial root commensals and light perception in Arabidopsis. This study applied a synthetic microbial community to reveal how low light directed on leaves induces shoot–root–microbiota modulation of the rhizosphere by bacterial communities, but not fungi or oomycetes. The study also found that microbial commensals promoted plant growth under low light conditions. However, this growth promotion came at a cost with reduced microbe-induced defense responses against foliar pathogens. This study highlighted an example of growth defense tradeoffs modulated directly by root microbial communities.

Integrating omics datasets across species can also help us understand crop microbiome community dynamics in relation to agroecosystem processes (Defrenne *et al.*, 2021). This is because crop and pathogen interactions function within a larger pathosystem. Therefore, integrated omics, for instance co-transcriptomic datasets, can help analyse host crops and pathogen interactions (Campos *et al.*, 2021; Gorshkov and Tsers, 2022). For instance, the study by Nobori *et al.* (2020, Preprint) utilized multi-omic datasets to decipher how plant immunity affects bacterial processes at the transcriptome and proteome levels. Similarly, Castrillo *et al.* (2017) investigated the connection between the phosphate starvation response and microbiome functionality and composition in Arabidopsis. Despite these efforts, it is still unclear how the association between microbial stress tolerance mechanisms and plant functional traits affects a plant’s performance under stress. To model global changes in plant and microbial associations with regard to agricultural processes, key relationships must be identified between plant and microbial traits. This may involve applying multi-omics approaches, advanced statistical methods, and mechanistic models to understand the key functional traits in microbes and plants, but also the trait trade-offs between roots, bulk microbes, endophytes, mycorrhizal fungi, and rhizosphere microbiota.

Incorporating the changes in the crop rhizosphere microbiome in response to abiotic and biotic stresses could be a powerful predictor in modeling approaches. Increasing evidence shows that the crop rhizosphere microbiome interactions can significantly improve our predictive power for soil suppressiveness of certain soil-borne plant pathogens (Schlatter *et al.*, 2017; H. Liu *et al.*, 2021; Zhou *et al.*, 2022). For example, using a semi-quantitative integration approach, Trivedi *et al.* (2017) identified bacteria belonging to *Acidobacteria*, *Firmicutes*, and *Actinobacteria* as key microbial predictors of agro-ecosystem soil suppressiveness against *Fusarium oxysporum*. This study highlights the fact that plant-associated microbial communities are less diverse compared with those in the surrounding environment (Fitzpatrick *et al.*, 2018; Carrión *et al.*, 2019; Gao *et al.*, 2021; Shalev *et al.*, 2022). This is driven by some microbes being adapted to colonizing plant tissues more than others, and the plant-selective recruitment of some microbiota (Waller *et al.*, 2005; Brader *et al.*, 2017; Sarkar *et al.*, 2019; H. Liu *et al.*, 2021). Notably, recent studies are revealing how plant roots launch successful defense responses against pathogenic microbes while permitting colonization by beneficial and commensal microbes (Sarkar *et al.*, 2019; Mahdi *et al.*, 2022). Using a synthetic bacterial community (SynCom), Teixeira *et al.* (2021), found that effective root colonization by commensal microbes is regulated by microbe-associated molecular patterns (MAMPs; MAMP-triggered immunity). They further found that MAMP-triggered immunity-suppressing commensal strains were efficient root colonizers compared with MAMP-triggered immunity-inducing strains. Other studies have found that in diseased soils, the rhizosphere microbiomes of different crops are directly regulated by root exudates (Du *et al.*, 2021). Rhizosphere microbiomes together with the environment form a sophisticated chemical network that harmonizes the plant microbiome (Fig. 2). In the future, we propose a rethinking of the traditional approaches used to analyse microbiomes in these soils to increase precision. This may involve using artificial intelligence (AI) strategies owing to their strong informative potential and potential to develop predictive models of the defense responses of plants and changes in their associated microbiomes (Antolín-Llovera *et al.*, 2012; Tian and Kong, 2022). AI platforms can be used to isolate large numbers of microbial taxa obtained from microbiome analyses through machine learning algorithms. For instance, Huang *et al.* (2023) proposed the novel concept of culturomics by automated microbiome imaging and isolation (CAMII) in human microbiome studies. This system uses machine learning approaches to automate the picking of bacterial colonies, maximizing morphological diversity and high-throughput identification of single colony bacterial genomic data. This system can be applied in plant microbiome studies to explore pathogenic and beneficial microbiota within the rhizosphere or endosphere environments. Therefore, deep learning and machine learning approaches hold immense potential in disease management such as in surveillance activities. These techniques are anticipated to enable future precision in monitoring the host response and alterations in the microbiome composition, for instance in the process of microbiome engineering (Hernández Medina *et al.*, 2022). Successful integration of AI pipelines and multi-omics approaches offers the promise of precise isolation and identification of diverse microbes from different samples.

Integrating omics can uncover new functions and features to identify vital interactions between crops and their native microbiomes that is applicable in plant breeding and the selection of better-performing crops (Nerva *et al.*, 2022b). This means that integrated omics can help determine dynamic functions within crop microbiomes by generating a picture of the expression, translation, and production of protein products during the course of plant–microbiome interactions (Zancarini *et al.*, 2021; Nerva *et al.*, 2022a). For example, a comprehensive study by Kwak *et al.* (2018) found the rhizosphere microbiomes of a resistant and susceptible tomato variety differed significantly. Using microbiomics and metagenomics integrated with host metabolomics, the study revealed that transplanting the rhizosphere microbiota from a resistant variety significantly suppressed tomato wilt disease caused by *Ralstonia solanacearum* in a susceptible variety. This study strongly supports the view that molecular mechanisms underlying plant disease resistance are strongly influenced by the genomes of both the host and its associated native microbiota (hologenome). Therefore, this shows that resistance can be inherited from one generation to the next and can benefit subsequent generations, a phenomenon referred to as ‘legacy inheritance’ (Yuan *et al.*, 2018). It is well known that numerous gene pools with desirable traits have been lost in different crops during domestication (Wagner, 2021). As such, exploiting introgression populations of wild species and domesticated varieties by using multi-omics approaches creates an opportunity to understand the genetic basis of lost traits and associated plant–microbe interactions in the transition from the wild species to the domesticated varieties. For example, transcriptomics and metabolomics approaches can identify genomic loci in host plants associated with transcripts and metabolites involved in plant microbiota modulation as well as pathogen defense (Wei *et al*., 2019; Szymański *et al.*, 2020; Wang *et al.*, 2022). Importantly, understanding microbiomes of the wild relatives of food crops using omics in combination with synthetic community approaches could enhance the designing of beneficial microbiomes towards increasing resilience to both abiotic and biotic stresses. Raaijmakers and Kiers (2022) propose rewilding ancestral microbiota in seeds and agricultural soils, and breeding crops with unique traits that support ancestral-microbe colonization. Through integrated omics approaches, the rewilding of ancestral microbiota in breeding programs will be precise and fast. By integrating metabolomics, metagenomics, and host genomics we can identify plant genetic loci involved in the recruitment of beneficial ancestral microbes and identify molecules released by plants that activate beneficial ancestral microbiota. This could provide beneficial microbes to enhance plant health and plant breeding. For instance, it has recently been shown that root-derived flavones were found to enrich bacteria in the *Oxalobacteraceae* taxon in the maize rhizosphere leading to enhanced growth and nitrogen acquisition (Yu *et al.*, 2021). Interestingly, Berlanga-Clavero *et al.* (2022) used transcriptomics and metabolomics and found that inoculation of melon seeds with *Bacillus subtilis* resulted in physiological and genetic changes in seeds, which altered the developmental status of germinated seeds leading to growth promotion and induced anti-fungal resistance. These multi-omics results represent key findings on how beneficial microbes alter the genetic responses in seeds, and the beneficial consequences of these alterations to the plants following germination.

As described, the combination of host and microbial datasets provides a unique approach to developing advances in plant–microbe interactions. Unfortunately, research gaps still exist in some niches; for instance few studies have incorporated viruses. Similarly, archaea and protists have also not been explored despite their significant contribution to microbiome diversity (Fig. 1D) (Trivedi *et al.*, 2020). Moving forward, it will be interesting to decipher the role of viruses, archaea, and protists in crop rhizosphere microbiomes in response to abiotic and biotic stresses. For instance, a review by Pratama *et al.* (2020) proposes genomics studies that can be used to decipher the effects of viromes on the plant rhizosphere microbiomes. Incorporating different omics approaches, for instance microbiological culturomics, along with cross-kingdom omics-based approaches could enhance the identification and construction of synthetic communities with beneficial traits in plants (Box 2). Previous studies have focused on bacterial communities, with finite reports on fungal, archaeal, protist, and viral communities (Fig. 1D) (Durán *et al.*, 2018; Hu *et al.*, 2020; Santos-Medellin *et al.*, 2021; Wolinska *et al.*, 2021). Fungal, protist, and viral communities can impact bacterial communities and host plants (Koskella and Taylor, 2018; Tedersoo *et al.*, 2020). In the future, studies should use integrated omics approaches to explore the role synthetic inter-kingdom microbial communities composed of bacteria, fungi, and even archaea, protists, and viruses play in contributing to plant growth and fitness. For instance, Zhou *et al.* (2022) used culturonomics and metagenomics to demonstrate how a synthetic community made up of bacteria and fungi maintained the health and suppressed Fusarium wilt disease in tomato plants. This study provided evidence that integrated omics approaches can be used to identify microbial traits that are vital for stress tolerance. Similarly, Durán *et al.* (2018) used members from three different microbial groups (oomycetes, bacteria, fungi) and found that bacterial biocontrol activities regulate cross-kingdom activities that maintain plant health in Arabidopsis roots. These studies provide evidence that integrated omics approaches can be used to identify microbial traits that are important for stress tolerance (Fig. 2).

In conclusion, as mentioned above, recent outbreaks of new pathogens and their spread to new regions accelerated by human activity and climate change have proved challenging to control. As such we suggest the utilization of multi-omics approaches in epidemiological studies to try and understand how plant pathogens spread. Integrating genomics and other omics tools in epidemiological studies, especially within emerging pathogens, seems like an onerous task. However, it has enormous potential to provide rich datasets that can be used to guide and inform policies in the agricultural sector. An example is the successful use of pathogenomic data created and shared by scientists to track the emergence of *Pyricularia oryzae* in Bangladesh (Kamoun *et al.*, 2019); these datasets enabled researchers to map out regions at risk of *P. oryzae* invasion and curb further spread.

## Technical advances in individual omics applied in plant microbiome studies

Over a decade, genomics technologies applying metagenomics and metatranscriptomics workflows have produced datasets that have helped to comprehend the complexity of plant microbial communities. Metagenomics offers novel insights into potential microbial community profiles and where necessary generates genomes that can be used as reference sequences within metatranscriptomics. Equally, metatranscriptomics is critical in providing gene expression levels. While post-transcriptional and post-translational gene expression can regulate protein synthesis, gene expression level enables microbes such as bacteria to adapt to any changes within the environment rapidly. As such, the application of metatranscriptomics has the potential to offer real-time regulatory plant–microbe interactions and other environmental response changes. Previously, studies involving diversity and functional soil microbial communities were hindered by the inability to culture many microorganisms in growth media. The development of culture-independent approaches has significantly increased the understanding of the biology associated with soil microbes. For example, genomic DNA, RNA, or metabolites can be extracted directly from soil samples and analysed through metagenomics, metatranscriptomics, metaproteomics, and metabolomics. Likewise, metabolomics provides a comprehensive picture of metabolic pathways that are involved in various species interactions and the underlying mechanisms of microbe and host interactions. This includes compounds secreted during beneficial interactions between plants and associated microbiota. It provides significant opportunities to study complex biological interactions within the rhizosphere and the responses between plants and microbes. It is an emerging field in the plant sciences that offers numerous opportunities to unravel the underlying mechanisms of plant–microbe interactions. However, in the study of beneficial plant–microbe interactions, it has not been fully utilized compared with other omics approaches such as metagenomics. The sections below discuss the application and opportunities of applying metagenomics, metatranscriptomics, and metabolomics as an integrative cohort for studying plant–microbe interactions towards improved crop productivity.

## Metagenomics

Metagenomics is the most utilized approach in the omics field. The advent of HTS technologies has vastly expanded our collection and understanding of plant-associated microorganisms (Lundberg *et al.*, 2012; Peiffer *et al.*, 2013; Ofek-Lalzar *et al.*, 2014). The availability of large-scale plant microbe genetic datasets has significantly advanced our knowledge of the molecular basis of plant–microbe interactions, including plant genotype and environmental factors that influence microbiome composition, structure, and evolution (Levy *et al.*, 2018). Studies have also shown that microbiomes are made up of many heritable taxa implying positive plant microbiome interactions during the evolutionary timeline (Peiffer *et al.*, 2013; Mousa *et al.*, 2016; Kong *et al.*, 2019). HTS datasets derived by targeted or shotgun sequencing have uncovered deeper information on the composition and function of core plant microbiomes, including the presence of disease-causing and beneficial individuals (Hu *et al.*, 2020;). For instance, Karasov *et al.* (2018), utilized HTS to carry out a multi-site, multi-year study of *Pseudomonas* species in Arabidopsis.

Similarly, Trivedi *et al.* (2016) utilized 16S rRNA sequencing, GeoChip 4.0 analysis, and qPCR techniques to evaluate the linkage between extracellular enzyme activity and microbial functional genes in soil microbial communities. This study revealed the differences in the activities of carbon-degrading enzymes were accurately predicted by functional gene abundance in soil microbial communities. Arguably, these findings could be applied as a predictor of soil carbon dynamics in different agricultural systems. Transposon sequencing (Tn-Seq) is another powerful tool that can be used to elucidate gene function in plant–microbe associations (Cole *et al.*, 2017). Tn-Seq involves mutating all genes in a genome using transposon insertions to determine their participation in a specified biological process (Cain *et al.*, 2020). Liu *et al.* (2018) utilized Tn-Seq and transcriptome sequencing to establish bacterial fitness in the Arabidopsis rhizosphere, using the plant-growth-promoting and biocontrol strain *Pseudomonas* sp. WCS365. The Tn-Seq approach can also be used to probe the genetics of root colonization in crops; for instance Sivakumar *et al.* (2019) utilized insertion sequencing (INSeq) to identify genetic determinants that contribute to the fitness of *Pseudomonas aeruginosa* PGPR2 in maize root colonization.

Genome-wide association studies (GWAS) are a powerful tool in the discovery of host plant quantitative trait loci corresponding with the microbiome richness, traits, and community structure in the phyllosphere and roots (Cole *et al.*, 2017; Demirjian *et al.*, 2023). GWAS and metagenome studies can be applied in exploiting the identification of specific microbial taxa and candidate plant genes, shaping the structure and function of the rhizosphere. Although the identification of primary plant loci involved in the regulation and recruitment of microbiota remains a challenge, recent studies have endeavored to do this (Bergelson *et al.*, 2019; Trivedi *et al.*, 2020; Escudero-Martinez *et al.*, 2022). Specifically, Escudero-Martinez *et al.* (2022) utilized a combination of metagenomics, host metabolomics, and transcriptomics to identify loci directly linked to the regulation of rhizosphere communities in domesticated and wild genotypes of barley. This study demonstrates the power of quantitative genetics in mapping out regions within the barley genome that are directly responsible for microbiota recruitment in the rhizosphere. Similarly, Oyserman *et al.* (2022) used a combination of quantitative plant genetics and microbiomics (metagenomics and amplicon sequencing) to identify rhizobacterial traits that are fundamental in microbiome assembly in tomato plants. The study mapped the putative molecular features of rhizosphere communities as quantitative traits in domesticated and wild tomato plants. Further, combining GWAS approaches with other omics techniques successfully identified specific loci that affect the phyllosphere microbiome. For instance, Wallace *et al.* (2018), used metagenomics and GWAS approaches and found that most quantitative trait loci for phyllosphere (leaf) microbiome traits in maize are of limited effect, making them undetectable. These results showed that, unlike in the rhizosphere environment where plant genetics plays a significant role in microbiota composition, the plant leaf microbiome composition is less impacted by host genetics and mainly impacted by stochastic events and environmental factors. Although GWAS does not specifically identify genes responsible for a given biological association, additional targeted re-sequencing approaches may be added to unravel the complexity (Deng *et al.*, 2021; Wang *et al.*, 2022). Recently, Shalev *et al.* (2022) used comparative genomics and annotation of GWAS hits to discover protective genes from *Pseudomonas* strains colonizing Arabidopsis roots. This study demonstrates the power of integrated omics and quantitative genetics to elucidate the complex environment of genotype and microbiome interaction, which could be applied in future microbiome-based plant breeding programs.

The genetic basis of plant–microbe interactions is quite complex, given the cross-kingdom nature of these interactions. Therefore, the need for multi-omics approaches is clear and more studies are needed to uncover the biological basis of symbiosis or pathogenicity of the plant-associating microbiota. Such integrative approaches offer an opportunity to pinpoint putative genes involved in plant–microbe interactions at a higher precision. Many genes associated with plant adaptation have been identified using multi-omics approaches in different plant-associated microbiota. Epigenomics approaches used for studying gene regulation are currently being proposed as a powerful tool that can be used to identify critical connections and biological functions in plant–microbe interactions (Ramos-Cruz *et al.*, 2021). Coupled with machine learning, epigenomics has the capacity to identify the differentially methylated regions of the plant genome during interactions with associated microbiota. For instance, Vigneaud *et al.* (2023) used epigenomics and transcriptomics approaches in understanding the interactions between poplar plants and their ectomycorrhizal fungus, *Laccaria bicolor*. They found that manipulation of the expression levels of two demethylase genes (*DML*) and a chromatin remodeler (*DDM1*) led to the host modulation of several parameters involved in poplar root colonization by *L. bicolor.* Interestingly, they found 288 transposable elements and 86 genes were differentially methylated between hypomethylated mutant lines and wild type poplar plants. These findings strongly suggest that host plants have the ability to remodel their associated fungal methylomes. This study is proof of principle in demonstrating the role the host plant’s epigenetic machinery plays during interactions with ectomycorrhizal fungi. It also raises the question of whether DNA methylation has a role in influencing plant interactions with endophytic fungi and bacteria. Despite these efforts, there is still a gap in elucidating the molecular mechanisms governing plant–microbiome associations at the community level. To close this gap, we propose an increase in studies utilizing pangenomic approaches in combination with other omics approaches to differentiate the pangenome sizes of plant-associated microbiota from non-plant microbiota.

## Metatranscriptomics

Metatranscriptomics is a powerful tool that captures the expression of genes at the community level, while transcriptomics is used to study the host transcriptome. Using transcriptomics, we are able to analyse the expression patterns of multiple transcripts in the hosts under different environmental set-ups using advanced methods (Chen *et al.*, 2023). Transcriptomics has been used to decipher plant–microbe interactions to understand the roles root exudates play in shaping the plant microbiome and shedding light on transcriptional temporal dynamics involved in host immune modulation, especially in biotic stress conditions (Hu *et al.*, 2018; Yuan *et al.*, 2018; Chai and Schachtman, 2022). On the other hand, metatranscriptomics approaches can be used to accurately identify active and passive microbial community members and quantify their expressed metabolic pathways (Nerva *et al.*, 2022a). Therefore, integrating metatranscriptomics with metaproteomics in plant–microbe interaction studies can improve our understanding of gene and protein function up to the species level (Turner *et al.*, 2013; Law *et al.*, 2022).

As mentioned, both metatranscriptomics and host transcriptomics approaches have transformed our ability to elucidate and explore transcriptional programs in host plants and microbial communities (Marquez *et al.*, 2019; Nobori *et al.*, 2022). They can capture vital aspects of the host transcriptome, shining light onto interactions between host processes and microbial community functions (Law *et al.*, 2022; Nerva *et al.*, 2022a). Previous studies have highlighted the physical mechanisms and molecular pathways by which microbes enter plants roots (Antolín-Llovera *et al.*, 2012; Bi and Zhou, 2017; Tian and Kong, 2022). Plant roots are made up of radial cell layers, and the functional impact of this concentric organization remains evasive (Salas-González *et al.*, 2021; Kawa and Brady, 2022). Fröschel *et al.* (2020) used translating affinity purification sequencing (TRAP-Seq) and RNA sequencing to analyse the specific cell type expression of labeled ribosomes. This approach allowed the extraction of ribosome-bound mRNA to acquire cell layer translatomes. Further, the authors also identified root cell layer responses that highlighted the distinct colonization strategies employed by these microbes. Also, the study showed that the vascular plant pathogen *Verticillium longisporum* significantly suppressed the synthesis of suberin and the endodermal casparian strip to successfully colonize the root tissues. This reaction was different from that for the beneficial endophyte *Serendipita indica*, which was localized in the cortex. This study illuminates the power of RNA sequencing and TRAP-Seq to decipher the plant root–microbe interactions leading to the development of useful strategies employed in crop improvement. These studies highlight the impact of host transcriptomics and metatranscriptomics approaches in elucidating crucial biological functions in plant–microbe interactions, for instance why some plant-associated microbiotas are better at colonizing their hosts than others (Gutleben *et al*., 2018).

The application of integrated metatranscriptomics can help distinguish passive and active microbiome community members and their expressed metabolic network pathways. For instance, integrated transcriptomics resulted in the discovery of the ‘soil memory’ phenomenon (Lapsansky *et al.*, 2016). This discovery uncovered that microbial communities convey in soil certain traits, including growth promotion, specificity, and heterogeneity, that can be inherited by the next generation (Lapsansky *et al.*, 2016; Raaijmakers and Kiers, 2022; Kong *et al.*, 2019). In particular, Li *et al.* (2019) used root metatranscriptomics and metagenomics to establish how past sowing significantly impacts the assembly and functions of the peanut rhizosphere microbial community. Elsewhere, a recent breakthrough utilized global transcriptomic responses in the root and leaf tissues of sorghum in response to drought over the growing season and found evidence of plant arbuscular mycorrhizal symbiosis disruption (Varoquaux *et al.*, 2019). The study found that critical genes involved in arbuscular mycorrhizal symbiosis were severely depleted in roots under drought conditions, in addition to the reduction of the (arbuscular mycorrhizal) fungal mass in roots. Genetic responses in the plant host correlated with the microbiome response, in this instance arbuscular mycorrhizal fungi, showing that drought constricts the symbiotic relationship between sorghum and arbuscular mycorrhizal fungi (Varoquaux *et al.*, 2019). Elsewhere, Maier *et al.* (2021) applied a combination of transcriptomics and metabolomics to reveal a novel molecular response termed the general non-self response (GNSR). They not only found a strong correlation between transcriptional and metabolic responses but also identified the presence of a potential link between GNSR and secondary metabolism, leading to the investigation of GNSR and specific metabolite features. GNSR involvement in abiotic and biotic stresses shows the untapped potential of plant microbiomes. Furthermore, this work highlights the potential and future application of integrated omics in plant microbiome interaction studies.

Challenges in plant microbiome studies include the identification and assignment of functions to individual microbiome communities or members (Sergaki *et al.*, 2018). Isolating specific microbiotas from plant–microbe studies to identify specific functions can be laborious especially when the phenotype cannot be identified (Houle *et al.*, 2010; Bodor *et al.*, 2020). Therefore, novel sensitive approaches are needed to characterize the plant microbiome beyond just the genus level. Through integrated omics approaches we can achieve an in-depth view of distinct taxa harboring vital functional traits. Viacava *et al.* (2022) used a combination of metatranscriptomics, metaproteomics, and metagenomics to identify and isolate arsenic-methylating microorganisms in anoxic soil from rice paddies. Such approaches can also determine how plant root microbiomes affect the above-ground induction of development. Equally, these approaches can be used to determine how the phyllosphere microbiome affects the response of the below-ground rhizosphere microbiome. This may significantly contribute to the development of synthetic communities that are able to induce defense responses in plants without negative implications for growth and development. Hou *et al.* (2021) applied a transcriptome approach to study root colonization by a synthetic community isolated from healthy plant roots. A key finding was the discovery of a transcription factor (MYC2) that coordinates a complex network between salicylic acid, gibberellic acid, and jasmonic acid to prioritize either microbiome-induced defense or growth depending on the available light conditions. Another example where co-transcriptome data support the drive for the development of robust plant and bacterial gene associations in plant–microbe interactions is the study by Nobori *et al.* (2022). The authors utilized a pipeline that allowed the investigation of host and microbial transcriptomes simultaneously during leaf colonization by a single bacterial strain. Notably, they identified enriched genes in the genome of the plant-associated bacteria that were significantly induced in plants, which highlighted bacterial adaptation to the plant leaf environment.

**Fig. 3.**
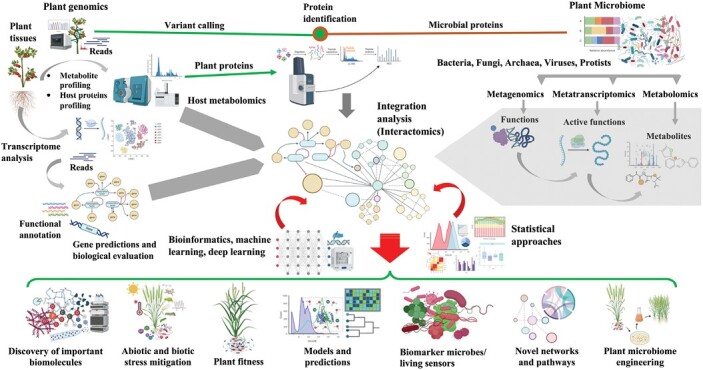
A representative integrative workflow demonstrating the use of omics datasets from plant hosts (left) and their corresponding microbiomes (right), integrating host and microbiome individual or combined omics datasets using statistical approaches or advanced bioinformatics and machine learning techniques to further elucidate the plant–microbiome interactions. These approaches can help to further increase our knowledge of plant microbiome gene functions in addition to identifying key traits and gene associations involved in plant–microbe interactions and functioning. The ultimate goals are improved plant fitness and performance, the discovery of novel molecules, an improved understanding of complex networks, and the creation of new models.

In the past few years, a number of innovative methods have been developed to contribute to understanding plant–microbe interactions. For instance, Haveman *et al.* (2021) utilized the MinION sequencing platform to perform a comparative analysis of lettuce microbiomes across temporal and spatial scales, to identify key drivers involved in plant growth and development. Such temporal and spatial scale comparisons will allow us to decipher how plant microbiomes change under environmental perturbations, and eventually contribute to improving plant health. In another comprehensive study, Mohammadi-Dehcheshmeh *et al.* (2018) used an integrative framework of machine learning, promoter analysis, and meta-analysis to unravel regulatory circuits and transcriptomic signatures that govern arbuscular mycorrhizal symbiosis in *Medicago truncatula*. This integrative approach identified key transcription factor genes involved in arbuscular mycorrhizal colonization including *MTR_6g029180*, *MTR_1g098300*, *MTR_5g031160*, *MTR_3g079620*, and *MTR_3g045440*. This study shows the potential of machine learning and models in elucidating omics datasets and the potential reduction of heterogeneity in experiments. Additionally, the integration of machine learning approaches with metagenomics and metatranscriptomics offers opportunities to develop novel microbial biomarkers that can be applied in ecological monitoring functions, for instance heavy metal accumulation in soil (Fig. 3) (Cheng *et al.*, 2023). Combining host and microbial information can provide an effective tool for developing hypotheses and advancing plant–microbe association studies, such as predicting the impacts of human activities and climate change within ecosystems. For example, Law *et al.* (2022) utilized both root transcriptomics and metatranscriptomics and found that hosts and their microbiomes can be catapulted by different environmental perturbations. They found that in response to limited nutrient availability, Norway spruce trees altered their fungal community structure by lowering sugar efflux and enhancing defense processes. This led to a pronounced restructuring of fungal communities in the rhizosphere by a decrease in basidiomycete species and a significant increase in ascomycete species. Additionally, a direct transcriptional link between fungal effectors and Norway spruce transcripts was discovered, giving molecular insights into this complex dialogue between host spruce trees and their fungal colonizers. These results linked the different mechanisms that are used by trees to limit the flow of carbon.

Integrating transcriptomics and metatranscriptomics approaches can be useful in understanding the potential effects of microbiomes on plant health (Maina *et al.*, 2018; Wamaitha *et al.*, 2018). Metatranscriptomes can be used to analyse different soil habitats to understand the role viromes play in maintaining plant health, either directly or indirectly. Through metatrascriptomics approaches, Starr *et al.* (2019) found that RNA viruses mostly colonize fungi in a grassland setting. This is particularly useful since some RNA viruses have been found to attack pathogenic fungi and cause them to revert to a non-virulent state (Koskella and Taylor, 2018; Pratama *et al.*, 2020; Zhang *et al.*, 2020; Koskella *et al.*, 2022). In conclusion, the studies described in this section provide important insights into the inner workings of complex plant microbiomes. We also foresee the integration of transcriptomics and metatranscriptomics will gain popularity in future studies, yielding interesting findings with agricultural applications.

## Metabolomics

Metabolomics is the study of small molecular products and intermediates of cellular metabolism. Plants release specialized metabolites that play key roles in ecology and coordinate interactions between plants and the environment. Even though microbes have different effects on plant fitness and growth, the mechanisms of how host plants recruit and assemble their microbiomes remain unclear. Deciphering the molecular pathways and factors underlying this crucial process will make a significant contribution to the field of microbiome engineering towards sustainable agricultural practices (Arif *et al.*, 2020; Albright *et al.*, 2022). Plants use approximately 20% of their synthesized carbon to produce root-derived molecules, which stimulate the formation of distinct root microbiota from the soil, a phenomenon referred to as the ‘rhizosphere effect’ (Stringlis *et al.*, 2018). As a result, the plant rhizosphere contains one of the richest and most diverse microbial ecosystems on Earth. Besides pathogens, the rhizosphere microbiome also contains beneficial microbes that stimulate plant health and/or promote growth (Hu *et al.*, 2018; Stringlis *et al.*, 2018; Di Lelio *et al.*, 2023). The exchange of metabolites between the microbiota and host plants is a key process that governs recruitment, communication, and modulation between these symbiotic partners (Geier *et al.*, 2020; Aharoni *et al.*, 2023). To maintain this organization, hosts and their microbiota use a wide range of metabolites that have specific localized functions within these associations.

Metabolites released by roots have been shown to selectively modulate the rhizosphere microbiome (Badri *et al.*, 2013; Stringlis *et al.*, 2018; Yuan *et al.*, 2018; Huang *et al.*, 2019; Koprivova *et al.*, 2019; Voges *et al.*, 2019). These metabolites consist of a wide range of low molecular weight compounds encompassing either primary metabolites (carboxylates, organic acids, sugars, amino acids, etc.) or secondary metabolites (coumarins, terpenoids, phenols, etc.) (Chai and Schachtman, 2022). These compounds act as nitrogen and carbon sources required for microbial growth. They also act as signaling molecules between hosts and their microbiota, thereby attracting, repelling, stimulating, or inhibiting microbial growth (Hu *et al.*, 2018). The composition and bioactive metabolite types vary significantly in different plant species. This is directly related to the rhizosphere microbial community composition. Therefore, under host-genetic control, root exudates are largely responsible for the assembly of specific rhizosphere microbial communities in different plant species (Baetz and Martinoia, 2014; Hu *et al.*, 2018). Root exudates play key roles in regulating the rhizosphere microbiota, facilitating interactions with beneficial microbes and suppressing pathogens. For instance, the breakdown of benzoxazinoid results in 2,4-dihydroxy-7-methoxy-2*H*-1,4-benzoxazin-3(4*H*)-one, which is a key secondary metabolite present in maize root exudates. It not only acts as a chemoattractant for beneficial plant growth-promoting microbes, but also as a strong allelochemical against neighboring plants and soil microbes (Neal *et al.*, 2012). In addition, another benzoxazinoid breakdown product, 6-methoxy-benzoxazolin-2-one, has also been found to confer resistance against a serious maize leaf pest (*Spodoptera frugiperda*) (Hu *et al.*, 2018). However, how the rhizosphere microbiome communities and functions respond to the release of chemical nutrients and exudates from host plants remains a fundamental question in plant microbiome studies. With advancements in metabolite profiling, metabolomics is currently playing a vital role in host–microbiome interactions and applied plant microbiome studies (Hu *et al.*, 2018; Geier *et al.*, 2020; Wolinska *et al.*, 2021; Getzke *et al.*, 2023). A comprehensive study by Huang *et al.* (2019) demonstrated that Arabidopsis plants produce specialized triterpenes that coordinate the assembly and modulation of Arabidopsis root microbiota. Using metabolomics, metagenomics, and mutant lines, the authors identified unique metabolic networks expressed specifically in the roots of Arabidopsis that are made up of different triterpene biosynthetic gene clusters. They used mutant lines that had triterpene biosynthetic gene clusters disrupted, and found that these harbored divergent microbiota compared with the wild type. Further, they isolated and purified triterpenes from Arabidopsis roots and found that these compounds selectively modulated the growth of 19 taxonomically divergent bacterial strains, originally isolated from Arabidopsis root microbiota. This study provides evidence of the direct roles root exudates play in the assembly of the root microbiota and opens opportunities for deploying integrated omics in microbiota engineering to improve plant growth and health. Elsewhere, Yuan *et al.* (2018) used a combination of host metabolomics and amplicon sequencing to study soil microbes that use chemical signals that are released by the microbes themselves or by plant roots to direct their growth in the direction of the host plant (Ye *et al.*, 2020). Therefore, by integrating metabolomics datasets from plants and their microbiomes we can probe whether plant and microbial metabolites have specific functions in different environmental conditions (Aharoni *et al.*, 2023; McRose *et al.*, 2023). Moreover, it has been widely reported that root exudates play key roles in the selection of the rhizosphere microbiome, and changing the root exudation patterns increases plant fitness (Wolinska *et al.*, 2021; Yu *et al.*, 2021; Getzke *et al.*, 2023). Therefore, by combining metabolomics and other omics approaches we can draw crucial correlations between metabolic findings and ecological observations. In particular, using metabolite fingerprinting coupled with shotgun metagenome sequencing, an antimicrobial coumarin was discovered to selectively modulate the rhizosphere microbial community in Arabidopsis (Stringlis *et al.*, 2018). This coumarin, scopoletin, was able to selectively inhibit the plant fungal pathogens *Verticillium dahlia* and *Fusarium oxysporum.* However, the induced systemic resistance-inducing and growth-promoting strains *Pseudomonas capeferrum* WCS358 and *Pseudomonas simiae* WCS417 were significantly tolerant of the antimicrobial effects of scopoletin. This study displays the potential of multi-omics data integration to pinpoint the precise molecular mechanisms involved in microbiome recruitment. The results were further confirmed by Voges *et al.* (2019), who utilized a combination of amplicon sequencing and metabolomics to affirm that coumarins are actively involved in shaping the root bacterial community in Arabidopsis. The scientific community has not yet fully unpacked the crucial roles that root exudates play in promoting plant fitness by modulating the plant-associated microbiota. The studies described in this section re-emphasize the role of integrated omics in understanding the functions of plant exudates in modulation of the rhizosphere microbiomes. Specifically, these approaches could inform the current gaps and questions: (i) Do specific metabolites in plant exudates take part in the recruitment of bacteria or fungi from specific phyla or genera? (ii) Under abiotic or biotic stresses, do specific metabolites in plant exudates help in the recruitment of specific microbes that are antagonistic to an attacking pathogen, or microbes that help plants to tolerate abiotic stresses? (iii) Does root colonization by microbes from different genera trigger the exudation of specific metabolites? (iv) During a pathogen attack, for instance, by foliar pathogens, do metabolites from the roots modulate the rhizosphere microbiome? Lastly, (v) during the early stages of a foliar pathogen attack, do specific metabolites help with the recruitment of microbes that can lead to induced systemic resistance?

Although plant exudates may recruit microbes that result in induced systemic resistance (Stringlis *et al.*, 2018), it is not clear how conditions change under biotic or abiotic pressure. Korenblum *et al.* (2020) addressed some of these questions by combining metabolomics, transcriptomics, and amplicon sequencing. Using systemically induced root exudation of metabolites (SIREM), they were able to show that different rhizosphere communities can induce distinct systemic changes in the root exudates of tomato plants. This study provides more insight into the role of rhizosphere communities in modulating secondary metabolites in local and systemic root–root signaling. This study led to further questioning of the role played by root exudates triggered by rhizosphere microbial communities between neighboring plant roots in defense priming and plant fitness. Using exometabolomics and metabolomics approaches, new molecular insights are being gained, particularly into how plants shape their microbial communities via root exudates (Huang *et al.*, 2019). Studies have shown that the metabolic content of microbiomes has a direct role in the ecosystem functioning of the mammalian gut (Gregor *et al.*, 2022). Could the case be similar for plant-inhabiting microbiomes? Integrating host metabolomics with microbiomics and microbial metabolomics could help us answer this fundamental question (Fig. 3). To decipher this, McRose *et al.* (2023) integrated host and microbial metabolomics and found that variations in the chemical microenvironment in the rhizosphere can significantly impact the effects of secondary metabolites in root exudates. They used redox-active metabolites from the model plant Arabidopsis (coumarins) and soil-dwelling pseudomonads (phenazines) to test this concept. The study showed that variations in pH and oxygen levels had direct effects on the capacity of phenazines and coumarins to promote the growth of pseudomonads. Interestingly, the capacity of coumarins and phenazines to increase the growth of these iron-limited pseudomonads was strongly influenced by pyruvate, succinate, and glucose, which are carbon sources readily found in root exudates. Therefore, the study concluded that plants modulate the released microbial secondary metabolites for their own benefit by altering the carbon content in root exudates. This study is a proof of concept showing the dynamic roles root exudates play in the rhizosphere microenvironment, in not only modulating the microbial community structure, but also its functions in regulating the roles of secondary metabolites released by the root microbiota. In addition to the role of metabolomics and microbiomics in studying ecosystem functioning, comparative genomics studies have shown metabolic links between microbial substrate utilization and plant exudation (Lu *et al.*, 2018; Kudjordjie *et al.*, 2019; Trivedi *et al.*, 2020). Such studies are assisting researchers in predicting functional and temporal patterns of community assembly in addition to understanding the complex interactions in the plant rhizosphere (Lu *et al.*, 2018; Kudjordjie *et al.*, 2019; Trivedi *et al.*, 2020). For instance, a study by Ye *et al.* (2020) found that exudates released by cucumber roots were able to attract a *Corallococcus* strain that directly predated on *Fusarium oxysporum* and modulated the soil microbial community. This subsequently reduced the effects of Fusarium wilt in both greenhouse and field experiments. Using integrated omics, this study was able to show that root exudates can attract microbes that directly predate on plant pathogens as well as modulate the root-associated microbiota for the benefit of the plant.

To further understand the complex and intricate interactions between plants and their associated microbiota, we propose a radical shift to spatial metabolomics of host–microbe interactions *in situ* and in real-time. This is because currently, most *ex situ*-based techniques are not able to link both the host’s and the microbiome’s metabolic phenotypes, considering metabolite identification requires homogenization, but the process of obtaining homogenates destroys or distorts the spatial organization of plant–microbe associations (Getzke *et al.*, 2023). Therefore, better techniques are needed to link plant–microbe and microbe–microbe interactions *in situ*, and this will provide more unbiased results when integrated with other omics techniques and help us understand rhizosphere changes over space and time. Mass spectrometry imaging can be used in combination with other techniques to visualize site-specific chemistry to create micrometer-scale maps of root or rhizosphere community metabolites. For instance, mass spectrometry imaging and microscopy successfully linked the distribution maps of metabolites to the location of corresponding microbes in a deep-sea mussel (Geier *et al.*, 2020). This study combined ionization mass spectrometry and fluorescence *in situ* hybridization to image the host–microbe metabolic interactions and symbiosis. This approach can be translated to plant–microbe association studies in combination with advanced phenomics and root imaging techniques (Rellán-Álvarez *et al.*, 2015; Fusi *et al.*, 2022) to elucidate *in situ* host–microbe metabolomics. Likewise, integrating novel omics approaches, for instance exometabolomics, with other omics approaches can help us understand microbe–microbe interactions and the roles these interactions play in microbiome establishment of different crops. For instance, Getzke *et al.* (2023) utilized exometabolomics and genetic approaches to identify novel antimicrobial compounds that are responsible for the strong antagonistic activity of *Pseudomonas brassicacearum* R401.

This section summarizes metabolomics applications in host and microbial studies to generate hypotheses that have enriched our understanding of plant–microbe interactions. However, there is still a lack of intricate and functional understanding of the plant–microbe-associated metabolomes. This is because root exudates can change unexpectedly in both quantity and composition during plant development and from tissue to tissue, resulting in differential recruitment of microbiota members. In addition, root exudate production and the structure and composition of plant microbiomes can drastically change as a result of seasonal changes and environmental factors. Further performing controlled plant–microbe interaction experiments does not give us an actual picture of the same interactions *in situ*, due to variation in results obtained from greenhouse and field studies. Taken together, these factors make deciphering molecular mechanisms involved in plant–microbe interactions a daunting task. To overcome these challenges, more multi-omics studies are needed to integrate not only classical omics approaches such as metabolomics, transcriptomics, proteomics, and metagenomics, but also new and emerging omics approaches, for instance genomics, epigenomics, and lipidomics. An example is the recently proposed ‘panomics’ approaches that integrates omics approaches such as epigenomics, proteomics, metabolomics, post-translational modifications, and system modeling in plant–microbe interactions studies. This will provide us with a systemic understanding of the sophisticated biological networks, increasing and expanding our biological knowledge base. Therefore, considering new improvements and developments in isolation, quantification, detection techniques, and bioinformatics platforms, integrating host and microbial metabolomics will play key roles in elucidating the complex molecular mechanisms and furthering our knowledge of plant–microbe interactions.

## Perspectives on genomic datasets

Given the technical advances in omics approaches and their wide application in plant microbiome studies, a plethora of genomic datasets are becoming available each year (Fig. 1). This presents an emerging challenge in the analysis and storage of HTS datasets that could be drawback in elucidating the hidden biological mechanisms driving key processes involved in plant–microbe interactions. New state of the art innovations must be developed and applied to uncover functional elements (for instance genes, proteins, and metabolites) and to eliminate noise signals. This mainly includes exceptional bioinformatics approaches to process the biological information obtained from such studies. Such approaches should be based on existing, trialed, and proven platforms that have shown success in host and microbial bioinformatics analysis. Currently, a multi-institute research project named RECONSTRUCT aims at combining and integrating reconstruction biology and omics-based *in silico* models in maize breeding to contribute to maize growth and fitness (Xie *et al.*, 2019). Another example is the maize functional genomics resource CUBIC (complete-diallel design plus unbalanced breeding-like inter-cross), which was successfully used to probe the genetic architecture of many vital agronomic traits in maize underpinning the maize microbiome interactions (Liu *et al.*, 2020).

To precisely compare datasets across different hosts, an integrated standardization of different dataset analyses will be needed. This should include controlled platforms to initiate community-driven standards of datasets. The Genomic Standards Consortium (GSC) (https://gensc.org/) is an example of an organization that aims to make genomic data more accessible. The GSC not only makes genomic data discoverable but enables efficient genomic data comparison and integration across the international community. GSC pioneered the establishment of minimum standards for characterizing sequence data information (MIxS: minimum (Glass *et al.*, 2014). MIxS standards comprise a registry that describes genomes (MIGS), metagenomes (MIMS), and marker genes (MIMARKS), providing minimum sequence information. In addition, the MIxS standards guide the interpretation of specific or distinct host-associated datasets and environmental datasets. These standardization efforts have added vital details and allowed quality analysis such as of phylogenetic relationships to create models in plant–microbe population evolution and dynamics (Garrido-Oter *et al.*, 2018; Karasov *et al.*, 2018). Host–microbe transcriptome analyses should be accompanied by relevant bioinformatics approaches, including suitable databases, for instance the one generated and maintained by the Plant-Associated Microbe Gene Ontology (PAMGO) consortium (Torto-Alalibo *et al.*, 2009).

Advances in AI could be applied in plant microbiome studies and help researchers in interpreting and processing the massive datasets coming from different omics platforms. This has the potential to provide new insights into plant–microbe interactions especially in food crops, leading to a deeper understanding of the plant microbiome associations. As highlighted in (Fig. 1A–D), huge multi-omics datasets are available every year, and machine learning approaches utilizing statistical methods can be used to identify patterns in these complex datasets. An example is EffectorP, a machine learning-based suite that contains tools used to predict pathogen effector proteins (Sperschneider *et al.*, 2016). Similarly, there is the recent development of an artificial intelligence network (AlphaFold) that determines protein 3D structures from amino acid sequences (Callaway, 2020). In addition, using AI-generated models we can elucidate how host plant traits are influenced by the microbiome and other factors than biotic or abiotic features (Fink *et al.*, 2023; Gonin *et al.*, 2023). Notably, any type of analysis should factor in potential gaps in understanding the differences in host cell density and host cell size, where variations can arise and interfere with the analyses of the microbial populations.

## Future perspectives and recommendations

Currently, there is persistent pressure for agricultural practices to meet the current demands for human nutrition, animal feed, and fiber. Unstable climatic conditions and other additional factors are among the main limiting factors that need to be addressed (Box 1). To address these challenges, a deeper understanding of genetic variation, cellular, developmental, and molecular pathways by which crops interact with their microbiomes in achieving stress tolerance, and crop fitness toward high productivity is needed. Studies focusing on the molecular mechanisms of plant–microbe interactions are progressively using multi-omics platforms. This has accelerated research in crop microbiome systems, which is providing a more reliable presentation of this biological phenomenon and is predicted to be a major driving force in the future agricultural systems. Additionally, the integration of plant- and microbe-focused omics approaches is providing large datasets that can be analysed to generate a mechanistic understanding and process-based agroecosystem models. These models can in turn present new strategies to enhance sustainable crop productivity. The global population is expected to reach 10 billion in 2050 (Zhao *et al.*, 2020). At the current pace, the world might miss its target to achieve the second Sustainable Development Goal (zero hunger) by 2030 (Global Hunger Index, https://www.globalhungerindex.org/). The COVID-19 pandemic has been a hindrance and has contributed to derailing food security efforts for many nations, especially developing countries, and this has caused significant negative effects on livelihoods and may continue to persist in the future.

This calls for future innovative strategies to improve our understanding of the mechanisms that enable plants to adapt to different environmental stresses and necessitates a deeper elucidation of plant–microbe interactions. This will be useful in the selection and breeding of crops that harbor beneficial microbiota to generate improved high-vigor crops (Arif *et al.*, 2020; Ke *et al.*, 2021; Albright *et al.*, 2022). Integrated omics offers the potential to design crops and microbes that interact more efficiently. Harnessing such technologies may include inter-kingdom studies to understand interactions between plants, microbes, nematodes, and insects. For instance, Di Lelio *et al.* (2023) utilized metabolomics and transcriptomics to show the interactions between the insect pest *Spodoptera littoralis* and tomato plants are strongly influenced by the tomato root colonizing biocontrol strain *Trichoderma afroharzianum.* This study found that rhizosphere microbiome members play a critical role in the control and regulation of plant–insect interactions. Such results contribute to future opportunities to explore the roles of microbial biocontrol towards sustainable agriculture.

The lack of appropriate approaches for studying plant–microbe associations *in situ* is hindering understanding of rhizosphere interactions; for instance knowledge of fungal and bacterial root attachment and colonization has been hindered by lack of appropriate approaches in studying plant–microbe associations. Notably, opportunities exist in integrating omics approaches with emerging novel approaches such as light sheet imaging, mesofluidics, and transparent soil in order to understand root–microbe interactions (van Schadewijk *et al.*, 2020; Borisjuk *et al.*, 2023). For instance, Downie *et al.* (2012) developed a porous substrate that can be used as a transparent soil in 3D imaging of root tissues and root-associated microbes. Elsewhere, Ge *et al.* (2022) developed an optical trapping approach that helped to reveal finer details of the attachment of the bacterial pathogen *Pectobacterium atrosepticum* on lettuce (*Lactuca sativa*) roots. Similarly, a study by Y. Liu *et al.* (2021) utilized three-dimensional live microscopy together with transparent soil to resolve plant–microbe interactions in lettuce plants *in situ* and in real-time. This approach allowed for precise tracking of the movement of *Bacillus subtilis* communities in the rhizosphere, revealing new patterns of activity. Despite these advances, moving forward, we recommend additional innovative methods such as combining three-dimensional live microscopy, transparent soil, stable isotope probing, and spatial metabolomics techniques to provide further information. In addition, the integration of high throughput phenotyping with different omics approaches will accelerate synthetic community development and promote microbial engineering. The integration of different phenomics approaches has revolutionized our understanding of crucial changes in plant physiology such as nutrient acquisition and water usage in relation to microbiome interactions (Chai *et al.*, 2021; Ferrocino *et al.*, 2023). Moreover, root anatomics is an emerging subset of phenomics that is underexploited yet holds promise for studying plant–microbe interactions (Strock *et al.*, 2022; Schneider *et al.*, 2023). Since rhizosphere microbiomes influence root traits, integrating root anatomics with other omics approaches, for instance transcriptomics, will help to uncover the role of rhizosphere microbiota in influencing plant traits. This will enhance tracking of the movement of microbes in the rhizosphere, as well as microbial and plant root metabolic changes in real-time.

Recently, application of system biology approaches in the study of plant–microbe interactions has gained a lot of interest. Computational modelling techniques in particular are being used to understand the hidden functions in metabolic networks by focusing on physical–chemical constraints (Kumar *et al.*, 2016). For instance, genome-scale models (GEMs) of metabolism provide valuable information on microbial communities. High-throughput GEM pipelines can be used in modeling to simulate spatial–temporal dynamics associated with microbial communities (Colarusso *et al.*, 2021), and spatial–temporal dynamics has been identified as a key bottleneck in plant–microbe interaction studies because microbiota associate in spatially structured communities that are densely packed (Monaco *et al.*, 2022). Further, species-level metabolic networks have the capacity to identify interaction types by metabolic scoring in microbe–microbe and host–microbial interactions (Kessell *et al.*, 2020). Creating computational models of plant–microbe interactions can greatly benefit from the incorporation of integrated multi-omics datasets to reduce the degree of degeneracy and improve resolution (Kumar *et al.*, 2016; Colarusso *et al.*, 2021). Computational models have significantly improved, integration of omics data and machine learning approaches will further develop this area. Such integration will further strengthen the predictive potential of computational models by dissecting multidirectional datasets to understand the complex relationships between phenomes and genomes in multi-omics datasets (Mishra *et al.*, 2019; Hernández Medina *et al.*, 2022). Additionally, incorporating transcriptomics, genomics, and metabolomics into metabolic reaction networks can help us identify condition-specific genes involved in host adaptation to environmental perturbations. For example, López *et al.* (2023) applied integrated omics and machine learning approaches to understand bacterial community assembly in plant rhizospheres. They integrated amplicon sequencing, metagenomics, and machine learning to identify key functional traits that are enriched in metagenome assembled genomes (MAGs) according to growth rate or niche. This study concluded that bacterial growth rate potential is the most significant genomic predictor for determining the ability of bacteria to colonize the rhizosphere. Elsewhere, Chien and Larsen (2017, Preprint) integrated flux balance analysis and different machine learning approaches (non-negative matrix factorization, artificial neural networks, and support vector machines) to decipher and forecast the ecological niche of plant-associated *Pseudomonas* species in the rhizosphere of trees. Finally, incorporating the discussed approaches from this review with CRISPR/Cas-mediated genome editing technology will foster new innovative ways to identify and characterize individual plants/crops or microbial candidate genes, focusing on their ability to transfer certain optimum amounts of nutrients from soils of distinct compositions to the plant. This could be a game changer and offer alternative routes in developing improved agronomic traits and sustainable cropping systems. Future studies should also focus on profiling and understanding the molecular mechanisms regulating plant microbial interactions in response to the relative abundance of multiple mineral nutrients. These efforts will benchmark potential innovations to support plant breeding, fertilizer formulations, biological application of beneficial microbes, and disease management to harness crop productivity through sustainable, innovative agricultural practices. Overall, this review has depicted and re-emphasized the current and future opportunities that omics approaches can offer to mitigate limitations in crop production especially triggered by environmental stresses, towards increasing global food productivity.
